# Targeted metagenomics using bait-capture to detect antibiotic resistance genes in retail meat and seafood

**DOI:** 10.3389/fmicb.2023.1188872

**Published:** 2023-07-13

**Authors:** Annika Flint, Ashley Cooper, Mary Rao, Kelly Weedmark, Catherine Carrillo, Sandeep Tamber

**Affiliations:** ^1^Bureau of Microbial Hazards, Health Canada, Sir Frederick Banting Driveway, Ottawa, ON, Canada; ^2^Research and Development, Ottawa Laboratory (Carling), Canadian Food Inspection Agency, Ottawa, ON, Canada

**Keywords:** antimicrobial resistance, gene detection, resistome, beef, chicken, oysters, shrimp, veal

## Abstract

Metagenomics analysis of foods has the potential to provide comprehensive data on the presence and prevalence of antimicrobial resistance (AMR) genes in the microbiome of foods. However, AMR genes are generally present in low abundance compared to other bacterial genes in the food microbiome and consequently require multiple rounds of in-depth sequencing for detection. Here, a metagenomics approach, using bait-capture probes targeting antimicrobial resistance and plasmid genes, is used to characterize the resistome and plasmidome of retail beef, chicken, oyster, shrimp, and veal enrichment cultures (*n* = 15). Compared to total shotgun metagenomics, bait-capture required approximately 40-fold fewer sequence reads to detect twice the number of AMR gene classes, AMR gene families, and plasmid genes across all sample types. For the detection of critically important extended spectrum beta-lactamase (ESBL) genes the bait capture method had a higher overall positivity rate (44%) compared to shotgun metagenomics (26%), and a culture-based method (29%). Overall, the results support the use of bait-capture for the identification of low abundance genes such as AMR genes from food samples.

## 1. Introduction

Antimicrobial resistance (AMR) is widespread throughout the bacterial kingdom. The mechanisms defining resistance are diverse, as are the genes encoding them. Some antibiotic resistance genes (AMR genes) such as those encoding transport proteins, stress response proteins, or regulators are encoded on the bacterial chromosome (Perry et al., 2014). Their resistance functions are secondary to their primary role in maintaining cellular homeostasis. Other AMR genes mediate resistance through the direct modification of the antibiotic or its cellular target. Often, these AMR genes are encoded on mobile genetic elements such as plasmids and are termed acquired resistance genes. From a public health perspective, acquired resistance genes are a concern because of their potential to mobilize between bacterial species (van Hoek et al., 2011). Such movement can explain the spread of AMR genes within and between multiple sectors including the environment, animals, and humans.

Food is an important vehicle that contributes to the global spread of AMR across borders and sectors (McDermott et al., 2002). Among food categories, AMR genes and AMR bacteria are most prevalent in meats and the use of antibiotics in animal husbandry has been linked to the presence of AMR genes and AMR bacteria in food animals (Nekouei et al., 2018; Carson et al., 2019). A number of national surveillance programs monitor AMR trends in food animals and meats have demonstrated the presence of clinically significant AMR genes in meats [for examples, see (Porter et al., 2020; Tate et al., 2021; Cox et al., 2022)]. The impact of these genes on human health however remains underappreciated due to the challenges associated with their detection in foods.

When considering the genomes of food cells and their associated microbiota, AMR genes represent a small fraction of the genetic content (Bengtsson-Palme et al., 2017). Of those AMR genes, acquired AMR genes are minor constituents, and are often present at levels below the sensitivity of direct detection methods (Randall et al., 2017; Peto et al., 2019). Successful detection of acquired AMR genes relies on their amplification to observable levels. This can be accomplished through enrichment, wherein the food sample is incubated in non-selective broth which permits bacterial recovery from injury and cell replication. Once bacterial cells concentrations reach a critical level they can be detected through plating onto selective agar, or via molecular assays such as PCR (Rao et al., 2021).

Whether targeting particular taxa (e.g., *Escherichia coli*) or resistance phenotypes (e.g., beta-lactam resistance), culture-based approaches offer a limited view of the diversity of foodborne antibiotic resistance genes. Metagenomics enables detection of all the AMR genes in the microbiome of food (the resistome) (Forbes et al., 2017). However, it is computationally and resource intensive especially for low abundance genes that require extensive deep sequencing (Bengtsson-Palme et al., 2017). To avoid repeated sequencing of off-target regions, targeted-sequencing approaches such as bait-capture have been described (Zhou and Holliday, 2012). Through the selective enrichment of AMR genes by hybridization to biotinylated probes prior to sequencing, bait-capture has been used to detect low abundance genes in stool, fecal, and wastewater samples at a reduced sequencing depth compared to shotgun metagenomics (Noyes et al., 2017; Lanza et al., 2018; Guitor et al., 2019). Here, we describe the use of a bait-capture approach to detect AMR genes in primary enrichments derived from retail meat and seafood samples. The results obtained by bait-capture are compared to those obtained by shotgun metagenomics sequencing as well as a previously published culture-based method targeting third generation cephalosporin (3GC) resistance and extended spectrum beta-lactamase (ESBL) genes. The feasibility of integrating a bait-capture approach to the routine monitoring of food surveillance samples is discussed.

## 2. Materials and methods

### 2.1. Baited library

**Figure 1:**
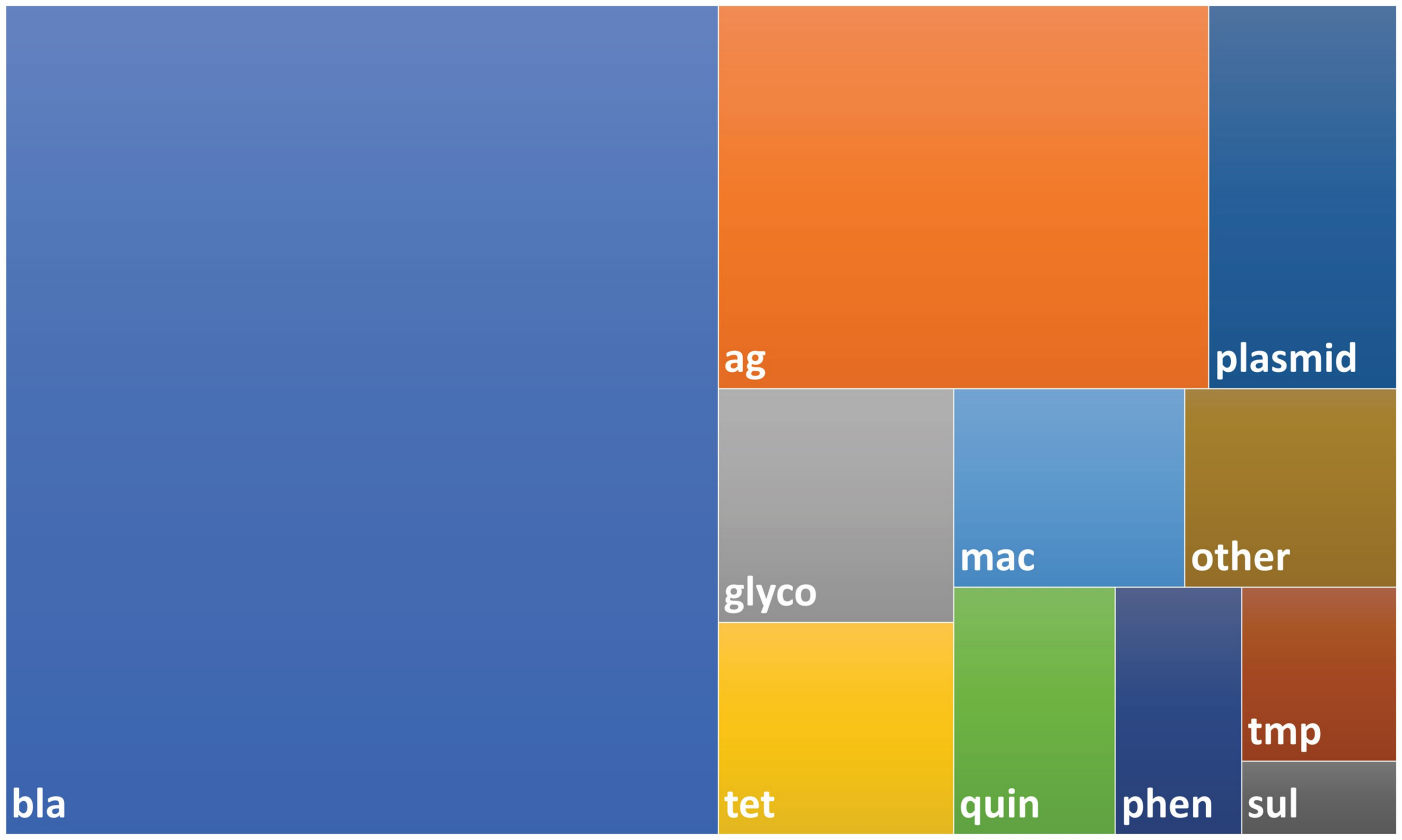
Treemap showing composition of bait library. The bait library consisted of 4,276 probes with 4,009 AMR genes and 267 plasmid genes. Probes were designed using sequences taken from the NCBI AMR, ResFinder and PlasmidFinder databases as described in the Methods. Abbreviations used for AMR gene classes: bla – beta-lactam, ag – aminoglycoside, glyco – glycopeptide, mac – macrolide, quin – quinolone, tet – tetracycline, phen – phenicol, tmp – trimethoprim, sul – sulfonamide.

The Resfinder (Zankari et al., 2012) (downloaded February 2017), Plasmidfinder (Carattoli et al., 2014) (downloaded February 2017), and NCBI Resistance Gene (Bioproject PRJNA31347, downloaded January 2017) databases were used to design a custom set of biotinylated oligonucleotide baits designed and manufactured by Arbor Biosciences (myBaits^®^, Ann Arbor, MI, United States). The completed set consisted of approximately 60,000 unique baits complementary to 4,276 unique gene sequences (Figure 1 and Supplementary File S1).

### 2.2. Food samples and primary enrichments

Primary enrichments from retail chicken, beef, and veal were prepared as described in Rao et al. (2021). Briefly, 25 g portions of retail chicken breast (*n* = 4), ground beef (*n* = 4), ground veal (*n* = 3 plus 1 technical repeat) were enriched overnight in modified tryptone soya broth (BD Difco) at 35°C. Seafood samples (live oyster shell stock, *n* = 2 and frozen shrimp, *n* = 2) were from on-going Health Canada surveillance programs. Seafood homogenates were prepared as previously described and incubated in buffered peptone water (BD Difco) overnight at 35°C. Target bacterial species (3GC resistant *Enterobacteriaceae*) were isolated after plating onto selective agar (ESBL Chromagar, Dalynn Biologicals). Isolates were screened by broth microdilution for resistance to cefotaxime and ceftriaxone, and the presence of beta-lactmase genes: *bla*_CMY-2_, *bla*_TEM_, *bla*_SHV_, *bla*_OXA_, *bla*_CTX-M1_, and *bla*_CTX-M9_ by PCR as previously described (Rao et al., 2021). Enrichments that harbored strains testing positive for either 3GC resistance or the presence of a target beta-lactamase gene were cryopreserved in 2X Brucella broth (Becton Dickinson) containing 30% glycerol and frozen at −20°C for short-term storage.

### 2.3. DNA extraction and microbial DNA enrichment library preparation

Ten milliliters of thawed food sample primary enrichments were centrifuged for 5 min at 6000 × *g* at 4°C and the pellet was resuspended in 400 μL of DNA/RNA Shield (Cedarlane). DNA was extracted using the Quick-DNA HMW MagBead Kit with RNAse A treatment according to the manufacturer’s protocol (Zymo Research Corp.). DNA was quantified using a Qubit fluorometer (Thermo Fisher Scientific). Microbial DNA was enriched using the NEBNext Microbiome DNA Enrichment kit (New England Biolabs) according to the manufacturer’s instructions and the Collect Enriched Microbial DNA protocol. Enriched DNA samples were purified using 1.8x AMPure XP beads following the AMPure XP Bead Cleanup protocol (Beckman Coulter).

### 2.4. Shotgun metagenomic library preparation and sequencing (unbaited)

Samples were fragmented using 2.5 μg of input DNA in 130 μL microTUBE snap-cap tubes using a M220 Focused Ultrasonicator (Covaris) to an approximate average fragment length of 700 bp according to manufacturer instructions. The DNA samples were further purified using a 0.8x AMPure XP (Beckman Coulter) cleanup. Illumina libraries were constructed using the NEBNext UltraII DNA Library Prep Kit (New England Biolabs) according to the manufacturer’s instructions using 500 ng input DNA and 5 cycles of PCR enrichment. Equimolar libraries were pooled and sent for paired-end Illumina NovaSeq 6000 (2 × 150 bp) sequencing at Genome Quebec.

### 2.5. Bait capture library preparation and sequencing

Construction of baited libraries was performed using the custom set of biotinylated probes and NEBNext shotgun metagenomics libraries according to the MYbaits manufacturer’s instructions (Daicel Arbor Biosciences). Hybridization of customized baits with 100 ng of metagenomics library was performed at 65°C for 20 h. Dynabeads MyOne Streptavidin C1 magnetic beads (Thermo Fisher Scientific) were used to isolate biotinylated DNA and KAPA HiFi HotStart Ready Mix (KAPA Biosystems) was used for amplification of bead-bound enriched libraries using NEBNext Unique Dual Index primers (12 cycles). The baited libraries were purified using a 0.8x AMPure XP (Beckman Coulter) cleanup, pooled in equimolar concentrations, and pair-end sequenced on a MiSeq instrument (v3 chemistry, 2 × 300 bp) according to manufacturer instructions (Illumina Inc.).

### 2.6. Bioinformatic analysis

#### 2.6.1. Read processing, downsampling, AMR and plasmid gene family identification

Raw Illumina reads were processed using FastP (v0.20.0) to remove adapter and barcode sequences, correct mismatched bases in overlaps, and filter low-quality reads (*Q* < 20) (Chen et al., 2018). The processed reads were randomly down-sampled per food commodity to the same read depth using seqtk (v1.3)^1^ and the sample command with default parameters and a seed of 31. AMR and plasmid gene families in each sample were identified using SRST2 [v0.2, (Inouye et al., 2014)] using the custom AMR and plasmid gene database used for bait design (default parameters). Gains achieved using bait-capture were calculated by dividing the percent baited mapped reads by the percent unbaited mapped reads from SRST2. Pearson correlations were calculated using GraphPad Prism v9.

#### 2.6.2. AMR gene family abundances and alpha diversity

AMR gene family abundances were calculated using the BAM output files from SRST2. BAM files were sorted by name using SAMtools [v1.7, (Danecek et al., 2021)] and the SAMtools sort function. Read counts were generated using HTSeq [v0.12.4, (Putri et al., 2022)] using the htseq-count function and parameters stranded = no and MapQ alignment quality of 0. Read counts were normalized to fragments per kilobase million. Statistically significant differences between unbaited and baited samples were determined using a Kruskal–Wallis test with a *post hoc* Dunn’s test with *p* < 0.001 considered significant. Alpha diversities were measured using Chao1 (AMR gene family richness) and Shannon (AMR gene family diversity) indexes using Phyloseq [v1.34, (McMurdie and Holmes, 2012)] (estimate_richness function using default parameters) and the HTSeq normalized read abundance data. Two-way ANOVAs with Sidak’s multiple comparison tests were used to determine statistical differences between shotgun metagenomic and enriched samples with *p* < 0.05 considered significant. Data were graphed using GraphPad Prism v6.

#### 2.6.3. AMR rarefaction

Illumina read data were randomly down-sampled in decreasing increments using seqtk (v1.3, see text footnote 1) and the sample command with default parameters and a seed of 31. AMR gene families at each read depth were identified using SRST2 [v0.2, (Inouye et al., 2014)] and the custom AMR gene database used to design the bait library (default parameters). Data were graphed using Graph Pad Prism v6.

### 2.7. Statistical analysis

Unless specified otherwise, statistical calculations were carried out using GraphPad Prism v9. Differences between the means of the unbaited and baited datasets were calculated using paired *t*-tests. Differences in means between commodities were calculated using unpaired *t*-tests or one way ANOVA with the Tukey’s *post hoc* test as deemed appropriate. *P* values less than 0.05 were considered significant.

### 2.8. Data availability statement

All SRAs are available in GenBank under BioProject ID PRJNA909287.

## 3. Results

### 3.1. Sequencing requirements for unbaited and baited libraries

**Figure 2:**
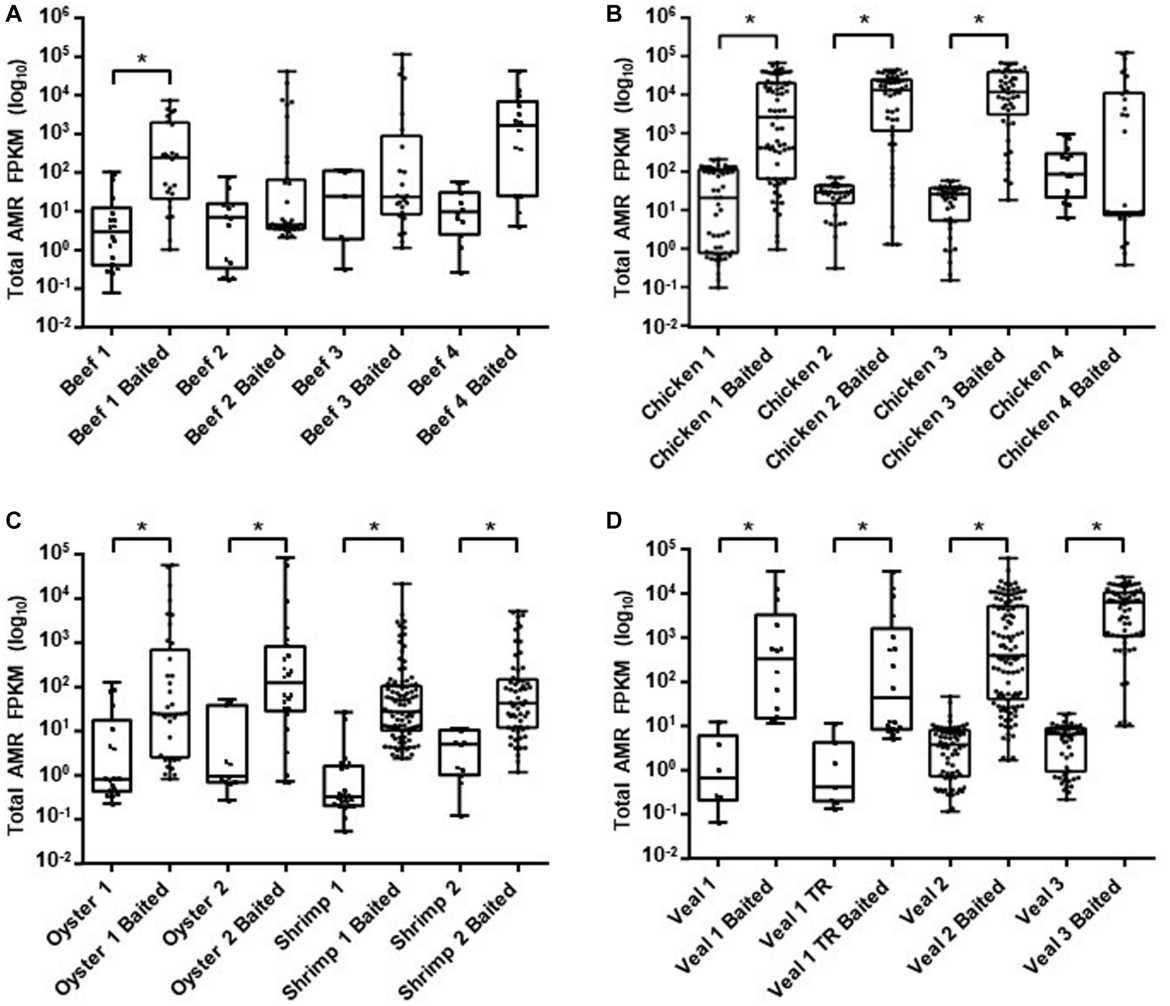
AMR gene family abundance. Raw read counts for ground beef **(A)**, chicken **(B)**, seafood **(C)**, and veal **(D)** unbaited and baited libraries were obtained from HTseq. Normalized read counts were calculated as fragments per kilobase million (FPKM) for gene families identified from SRST2 analysis. Horizontal lines represent the median, boxes indicate the inter-quantile range and whiskers represent values within 1.5 IQR of the lower and upper quantiles. Asterisks denote statistically significant differences (*p* < 0.001) using a Kruskal Wallis test.

To compare sequence-based detection methods for AMR genes in retail meats, two sets of sequencing libraries were prepared from overnight enrichment cultures of retail beef, chicken, oyster, shrimp, and veal. One library was prepared from the total DNA fraction isolated from each enrichment culture (unbaited) and the other from a subset of DNA that had been subjected to bait-capture to target AMR and plasmid genes. On average 304 million paired end reads per sample were obtained from the unbaited libraries and 9.2 million reads per sample for the baited libraries. After subsampling to the same read depth per food commodity, a greater proportion of baited reads mapped to genes within the AMR and plasmid databases (*p* < 0.0001, and *p* = 0.0003 respectively, Table 1). Compared to the unbaited dataset, the gains achieved with bait-capture ranged from 115–2280 X for AMR genes and 89–570 X for plasmids. The highest gains were observed in those samples with the fewest unbaited reads mapping to the AMR database (Pearson *r* = −0.58, *p* = 0.0180). The majority of samples in the baited dataset (12/16) had significantly higher AMR gene fragments per kilobase million (FPKM) compared to their unbaited counterparts (Figure 2). The remaining samples were either trending toward an increase (Beef 4) or had a high proportion of low abundance fragments identified (Beef 2, 3, Chicken 4). Collectively, the increased AMR gene content of the baited dataset resulted in approximately 2-fold more detections of AMR gene family classes, AMR gene families, and plasmids in the baited dataset compared to the unbaited set (Table 2). The highest increases were observed with the seafood samples (shrimp for AMR genes, and oysters for plasmid genes).

**Table 1:** Sequence read mapping to AMR gene and plasmid databases.

Sample	Raw reads (M)		Subsampled read depths (M)		AMR database (% reads mapped)			Plasmid database (% reads mapped)		
	Unbaited	Baited	Unbaited	Baited	Unbaited	Baited	Gain	Unbaited	Baited	Gain
Beef 1	337	14.4	282	7.1	0.22	25.4	115 X	0.04	4.66	117 X
Beef 2	291	7.5	282	7.1	0.22	36.1	164 X	0.08	24.8	310 X
Beef 3	285	8.5	282	7.1	0.06	70.4	1173 X	0.01	4.03	403 X
Beef 4	290	7.3	282	7.1	0.21	46.1	220 X	0.05	28.5	570 X
Chicken 1	328	15.1	315	8.7	0.24	67.0	279 X	0.17	17.3	102 X
Chicken 2	401	9.7	315	8.7	0.14	60.0	429 X	0.08	17.5	219 X
Chicken 3	367	11.0	315	8.7	0.11	52.0	473 X	0.13	15.9	122 X
Chicken 4	318	8.8	315	8.7	0.52	79.9	154 X	0.07	11.0	157 X
Oyster 1	276	11.4	275	11	0.16	40.4	253 X	0.01	0.89	89 X
Oyster 2	306	11.3	275	11	0.08	57.9	724 X	0.00	0.14	NC
Shrimp 1	300	6.9	168	3.2	0.09	24.0	267 X	0.00	6.58	NC
Shrimp 2	170	3.3	168	3.2	0.02	10.7	535 X	0.00	3.49	NC
Veal 1	274	6.9	257	6.8	0.01	26.3	2630 X	0.00	5.82	NC
Veal 1TR	360	8.0	257	6.8	0.01	28.0	2800 X	0.00	6.61	NC
Veal 2	259	81	257	6.8	0.08	60.0	750 X	0.04	17.7	443 X
Veal 3	316	9.4	257	6.8	0.04	40.3	1008 X	0.00	6.15	NC

TR, technical repeat; NC, non-calculable.

**Table 2:** Number of AMR gene family classes, AMR gene families, and plasmids detected in unbaited and baited libraries.

Sample	AMR classes		AMR gene families		Plasmid genes	
	Unbaited	Baited	Unbaited	Baited	Unbaited	Baited
Beef 1	7	9	18	24	21	34
Beef 2	5	10	13	36	23	36
Beef 3	3	8	7	25	5	25
Beef 4	5	6	11	14	24	31
Chicken 1	14	14	54	71	39	48
Chicken 2	9	11	36	49	25	27
Chicken 3	9	9	41	45	32	35
Chicken 4	6	9	14	29	21	24
Oyster 1	6	10	18	37	3	17
Oyster 2	4	7	11	24	3	13
Shrimp 1	9	17	22	99	16	47
Shrimp 2	6	15	11	61	15	40
Veal 1	3	5	6	14	14	19
Veal 1TR	3	6	7	18	14	22
Veal 2	12	16	70	108	48	68
Veal 3	9	9	55	65	22	34
Total	103	162	394	719	325	520

TR, technical repeat.

**Figure 3:**
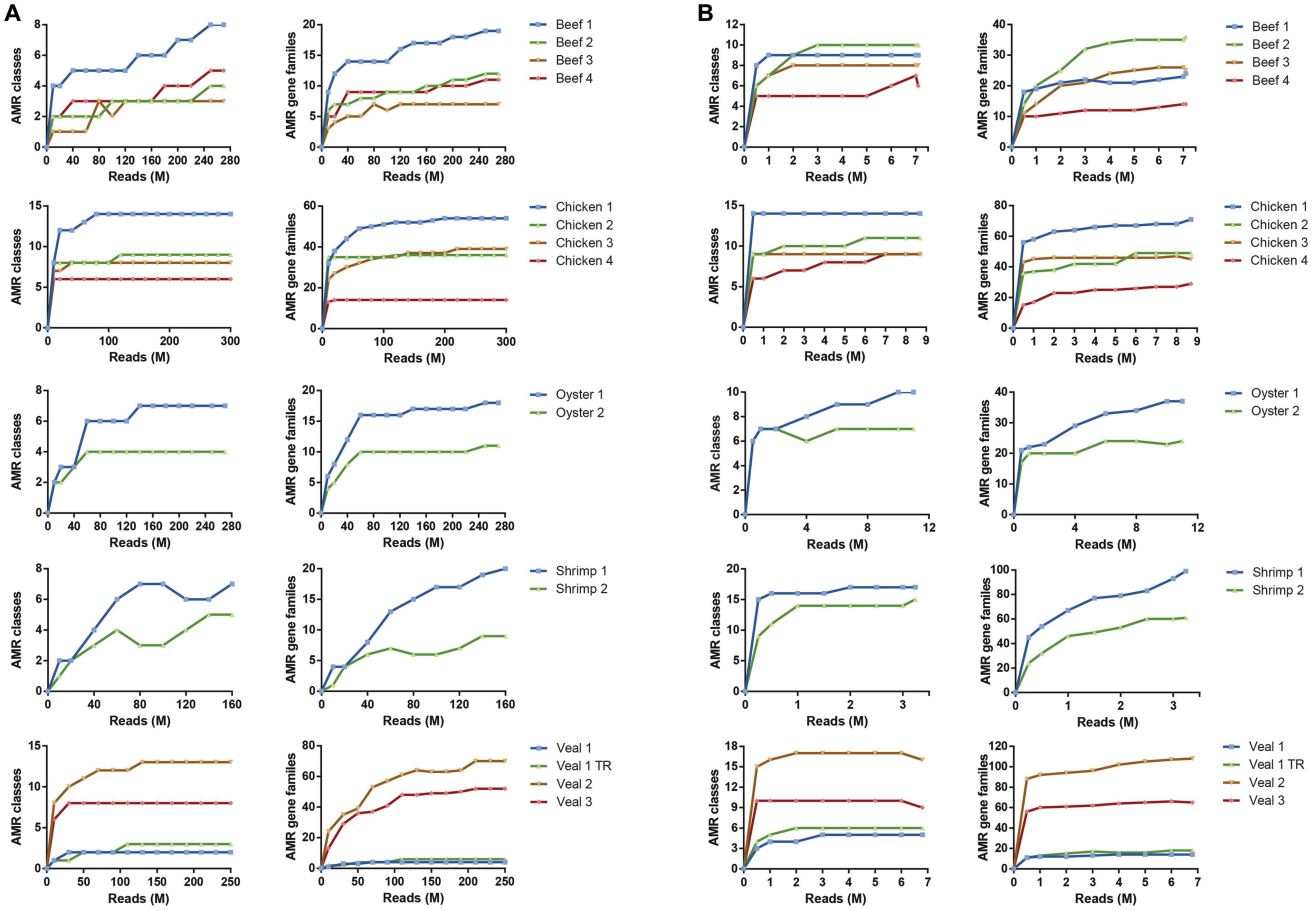
Read depth required to detect AMR gene family classes and AMR gene families. Rarefaction curves of unbaited **(A)** or baited **(B)** libraries for AMR gene family classes (left) and AMR gene families (right). Reads were randomly subsampled at specific intervals and AMR classes identified by SRST2 analysis. Sequencing depth is shown as million reads (M).

The number of AMR classes and AMR gene families detected increased with increasing sequencing depth until reaching saturation in most samples. In total 20 AMR classes and 196 AMR gene families were identified in the 16 samples (Supplementary Table S2). The number of reads required to reach saturation for identification of AMR classes and gene families was variable for each commodity, but in general 25–50-fold fewer reads were required to reach saturation with the baited dataset (overall average = 3.2 ± 2.8 M reads for AMR class and 6.7 ± 2.2 M reads for AMR gene family) compared to the unbaited dataset (average = 120 ± 79 M reads for AMR class and 171 ± 70 M reads for AMR gene family) (Figure 3). Similarly, the range of reads required to reach saturation was much smaller for the baited dataset (AMR class: 0.5–10 M reads; AMR gene family: 3.2–11 M reads) compared to the unbaited dataset (AMR class: 10–250 M reads; AMR gene family: 20–250 M reads). Some samples, particularly from the unbaited beef libraries, did not reach a plateau, and required significantly more reads to approach AMR class saturation compared to the other meat types [218 M reads (beef) vs. 120 M reads (all other meat types), *p* = 0.0354]. No other relationships were observed between the minimum number of reads required to reach saturation and meat type or number of AMR classes/gene families identified in each sample. No trends were observed with respect to AMR class/gene family identification and required number of reads.

### 3.2. Resistome comparison

**Figure 4:**
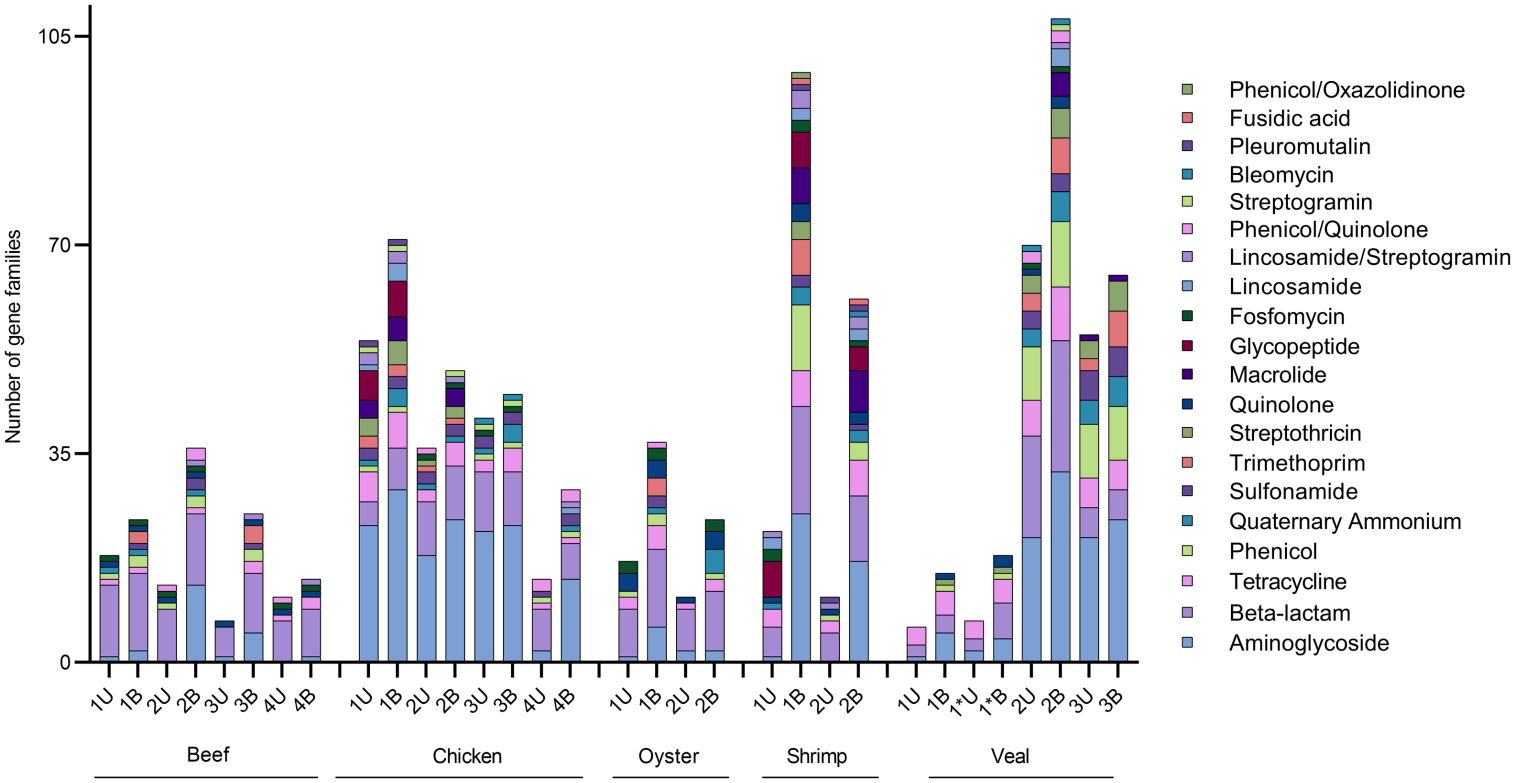
Absolute quantification of AMR gene families in retail meat primary enrichments as identified in unbaited (U) and baited (B) datasets. AMR gene families were identified using SRST2 and tabulated/visualized using GraphPad Prism v9. Colored segments of each stacked bar, if present, correspond to the number of gene families mediating resistance to the antibiotic classes as listed in the legend from aminoglycoside (bottom) to phenicol/oxazolidinone (top). * technical repeat of veal sample 1.

Both unbaited and baited datasets yielded 20 AMR classes. Genes for beta-lactam, aminoglycoside, and tetracycline resistance were the most prominent with detections in every sample (Supplementary Table S1). Genes for phenicol, quaternary ammonium compound, and sulfonamide resistance were the next most frequently detected classes. Some differences in AMR gene content were noted between commodities. On average, the shrimp samples had the highest number of detections (80), followed by veal, chicken, oyster, and beef (52, 49, 31, and 25 AMR genes respectively). Aminoglycoside resistance was the most abundant resistance class detected in shrimp, veal, and chicken, whereas beta-lactam resistance was the most frequently detected class in the beef and oyster samples (Figure 3). The remaining AMR classes were represented in varying degrees in the different meat commodities. Fusidic acid resistance genes were uniquely detected in the shrimp samples and these samples also had significantly higher rates of detection of macrolide resistance genes (*p* = 0.0004). There was a notable absence of quinolone resistance genes in chicken (Supplementary Table S1). Apart from these observations, the differences in AMR gene content among commodities were not significant due to variability among individual samples within each commodity (e.g., compare Chicken 1 and 4; Veal 1 and 2, Supplementary Table S1, Figure 4).

**Figure 5:**
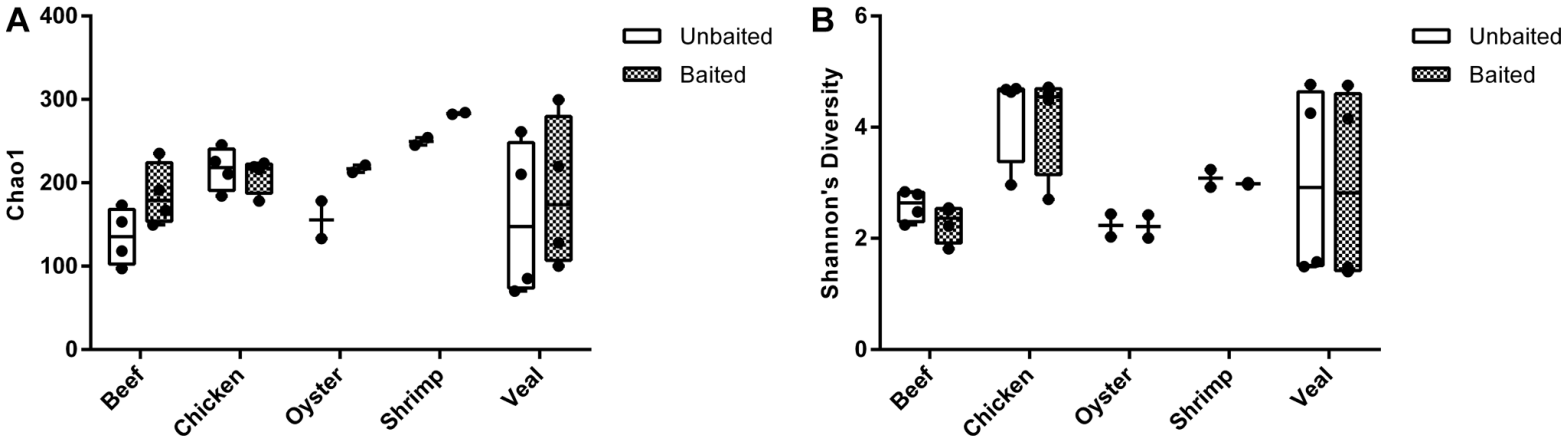
Alpha diversity of unbaited and baited beef, chicken, oyster, shrimp, and veal samples. **(A)** Chao1 gene family richness (estimated # of gene families) and **(B)** Shannon’s Diversity indices (gene family evenness). Horizontal lines represent the median, boxes indicate the inter-quantile range and whiskers represent values within 1.5 IQR of the lower and upper quantiles. Alpha diversities were calculated using PhyloSeq in R and raw normalized read abundances per sample.

The number of AMR gene family classes, AMR gene families, and plasmid genes detected were higher or equivalent using the baited dataset compared to the unbaited set (Table 2). Per sample, an average of 10 ± 4 AMR classes (range: 5–17) and 45 ± 29 AMR gene families (range: 14–108) were identified using the baited libraries compared to 7 ± 3 AMR classes (*p* = 0.0003) (range = 3–14) and 25 ± 20 AMR gene families (*p* = 0.0001) (range = 6–70) using the unbaited libraries (Table 2). Analysis of the gene family richness between the baited and unbaited library sets did not reveal any significant differences. Similarly, the evenness of the gene family distribution was equivalent between baited and unbaited libraries (Figure 5). With respect to the ranking of the resistance classes within meat commodities, there was good agreement between the baited and unbaited library sets when the relative abundance was relatively high (Supplementary Table S2). For example, among the chicken samples, the four most abundant AMR classes were aminoglycoside, beta-lactam, tetracycline, and sulfonamide in both datasets. The fifth and sixth most abundant classes, however, were quaternary ammonium compounds and macrolides (baited) vs. glycopeptide and streptothricin (unbaited).

### 3.3. Plasmidome comparison

**Figure 6:**
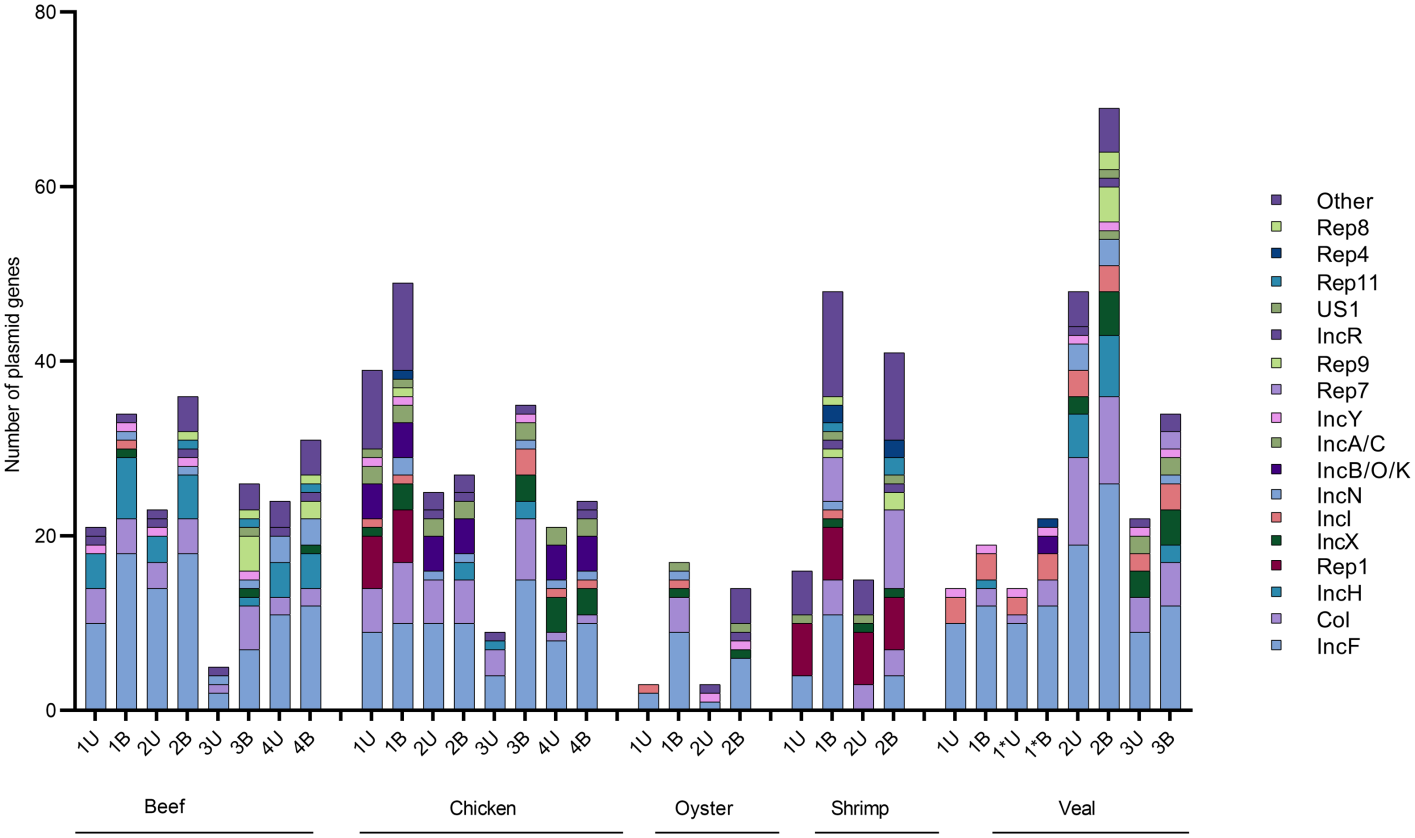
Absolute quantification of plasmid genes families in retail meat primary enrichments as identified in unbaited (U) and baited (B) datasets. Plasmid genes were identified using SRST2 and tabulated/visualized using GraphPad Prism v9. Colored segments of each stacked bar, if present, correspond to the number of plasmids genes belonging to the replicons as listed in the legend from IncF (bottom) to Other (top). * technical repeat of veal sample 1.

Using the unbaited dataset, we were able to detect 302 plasmid genes belonging to 35 replicon types within the retail meat preenrichment samples. The baited dataset contained 520 plasmid genes and 37 replicon types. There was quite a bit of variation in the number of plasmids detected within samples of the same commodity. On average, veal and chicken had the most plasmid detections in the unbaited dataset (24 and 25 plasmid genes respectively) and oysters had the least (3 plasmid genes) (Table 2). In the baited set the shrimp samples had the most plasmids detections (average = 44) and oysters had the least (average = 15). Across commodities, IncF was the most frequently detected plasmid replicon followed by Col, IncH, IncX, and Rep 1 (Figure 6). There was a higher association of Rep and US type plasmids in shrimp than in other commodities (Rep 1, 7, 4, 10b, 22, all *p* < 0.0500). In chicken, there was a higher rate of detection for IncA/C (8 detections compared to 0, 1, 0, and 3 for beef, oyster, shrimp, and veal, *p* = 0.0025) and IncB/O/K (12 detections compared to 0, 0, 0, and 2 for beef, oyster, shrimp, and veal, *p* = 0.0208) compared to other commodities.

A high correlation was observed between the number of AMR genes and number of plasmids detected in a sample (Pearson *r* = 0.85, *p* < 0.0001). With respect to individual replicon types, this relationship held for Col (*r* = 0.67, *p* = 0.0042) and IncX (*r* = 0.59, *p* = 0.0160). Similarly, twelve of the twenty AMR gene family classes were positively correlated with the presence of plasmid genes (Table 3).

**Table 3:** Relationship between AMR classes and plasmid genes detected in retail meat preenrichments.

AMR class	*r* ^a^	*P*
Fusidic acid	0.98	0.0043
Glycopeptide	0.88	0.0019
Pleuromutilin	0.85	0.0072
Lincosamide	0.84	0.0011
Aminoglycoside	0.75	0.0007
Streptothricin	0.71	0.0100
Tetracycline	0.69	0.0032
Macrolide	0.68	0.0203
Phenicol	0.67	0.0049
Beta-lactam	0.63	0.0083
Lincosamide/Streptogramin	0.54	0.0390
Trimethoprim	0.53	0.0407

a, Pearson correlation coefficient (r).

### 3.4. AMR gene content of baited and unbaited libraries

Collectively among the 16 retail meat samples, there were 733 identifications of AMR gene families. Just over half of the detections (380, 51%) were present in both the baited and unbaited data sets (Table 4). Three hundred thirty-nine additional detections (47% of the total) were made using the baited dataset alone. These detections spanned all 20 AMR classes with the majority belonging to the aminoglycoside (107/339) and beta-lactam resistance (57/339) classes. Among the meat commodities the shrimp samples had the least overlap between the baited and unbaited sets (19% of identified genes) with the majority of detections occurring in the baited dataset alone (80% of identified genes). The chicken samples had the most overlap (68%) with only about a third of detections occurring in only the baited data set.

**Table 4:** Unique AMR gene families identified via total and targeted sequencing.

Sample	Genes families identified (%)		
	Both	Baited	Unbaited
Beef 1	16 (61%)	8 (31%)	2 (8%)
Beef 2	12 (32%)	24 (65%)	1 (3%)
Beef 3	7 (28%)	18 (72%)	0
Beef 4	10 (67%)	4 (27%)	1 (7%)
Chicken 1	54 (76%)	17 (24%)	0
Chicken 2	35 (70%)	14 (28%)	1 (2%)
Chicken 3	39 (83%)	6 (13%)	2 (4%)
Chicken 4	13 (43%)	16 (53%)	1 (3%)
Oyster 1	16 (41%)	21 (54%)	2 (5%)
Oyster 2	11 (46%)	13 (54%)	0
Shrimp 1	21 (21%)	78 (78%)	1 (1%)
Shrimp 2	10 (16%)	51 (82%)	1 (2%)
Veal 1	6 (43%)	8 (57%)	0
Veal 1_TR	7 (39%)	11 (61%)	0
Veal 2	68 (62%)	40 (36%)	2 (2%)
Veal 3	55 (85%)	10 (15%)	0
Total	380	339	14

There were fourteen instances (2% of total detections) where genes were identified in the unbaited dataset of some samples but not in the corresponding baited dataset. These fourteen instances were observed in all meat types and were attributed to eight genes (*bla*_CFE_, 4 instances, *bla*_LEN_, 2 instances, *bla*_OHIO_, 2 instances, *oqxB*, 2 instances, *bla2*, once, *fosA*, once, *mexX*, once, and *bla*_ORN_, once). Multiple alleles of these genes were present in the bait probe library, except for *bla*_CFE_, *bla*_OHIO_, and *mexX*, which were present only as single copies. All eight genes except *bla*_LEN_, *bla2*, and *mexX* were detected at least once in the baited dataset of other samples.

### 3.5. Detection of specific AMR genes

**Figure 7:**
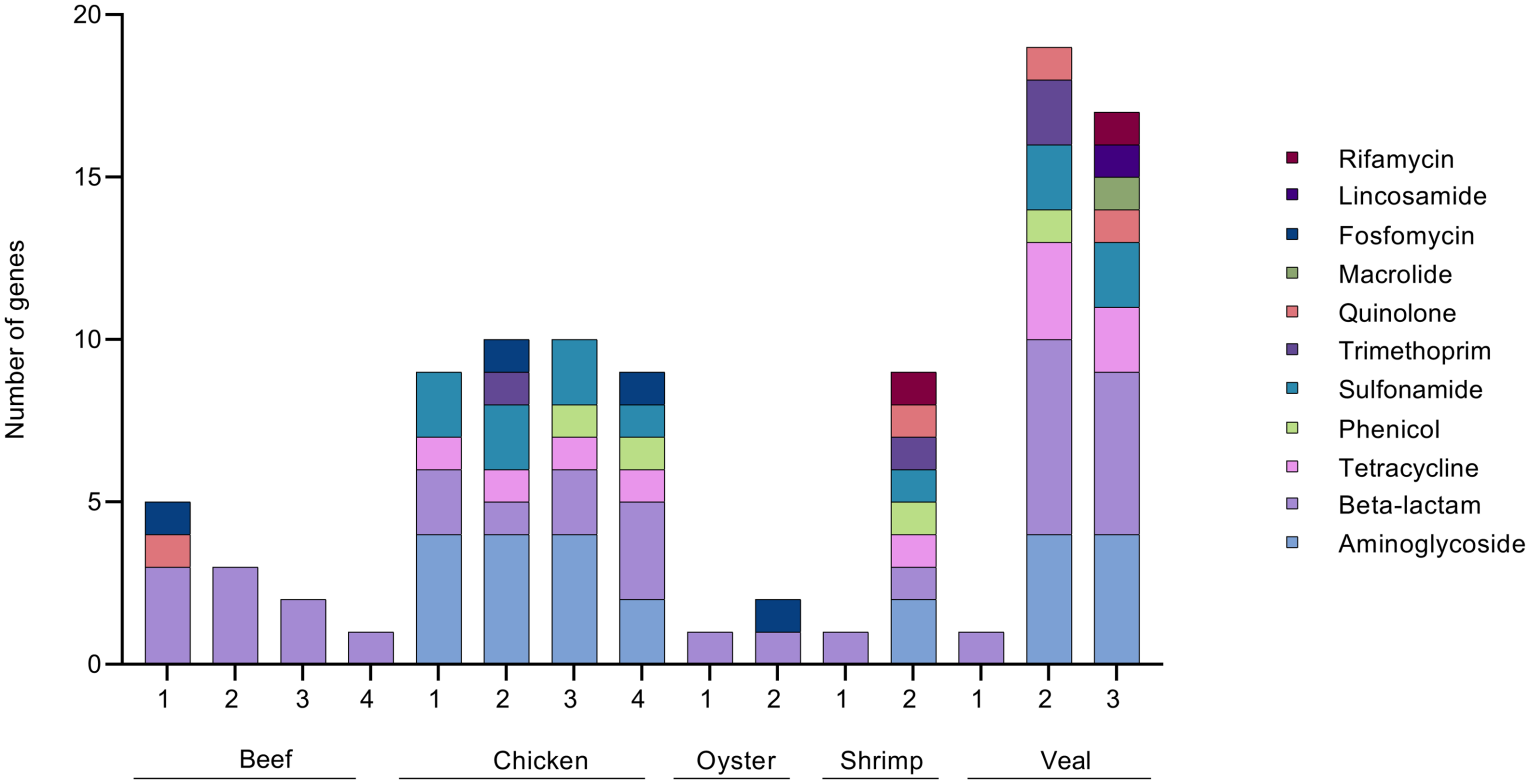
ARG profile of 3GC resistant *Enterobacteriaceae* strains. 3GC resistant strains were isolated from retail meat primary enrichments by plating on CHROMagar ESBL. Isolates were sequenced and AMR gene sequences identified using ResFinder 4.0 and tabulated/visualized using GraphPad Prism v 9. Colored segments of each stacked bar, if present, correspond to the number of genes mediating resistance to the antibiotic classes as listed in the legend from aminoglycoside (bottom) to rifamycin (top). * technical repeat of veal sample 1.

**Figure 8:**
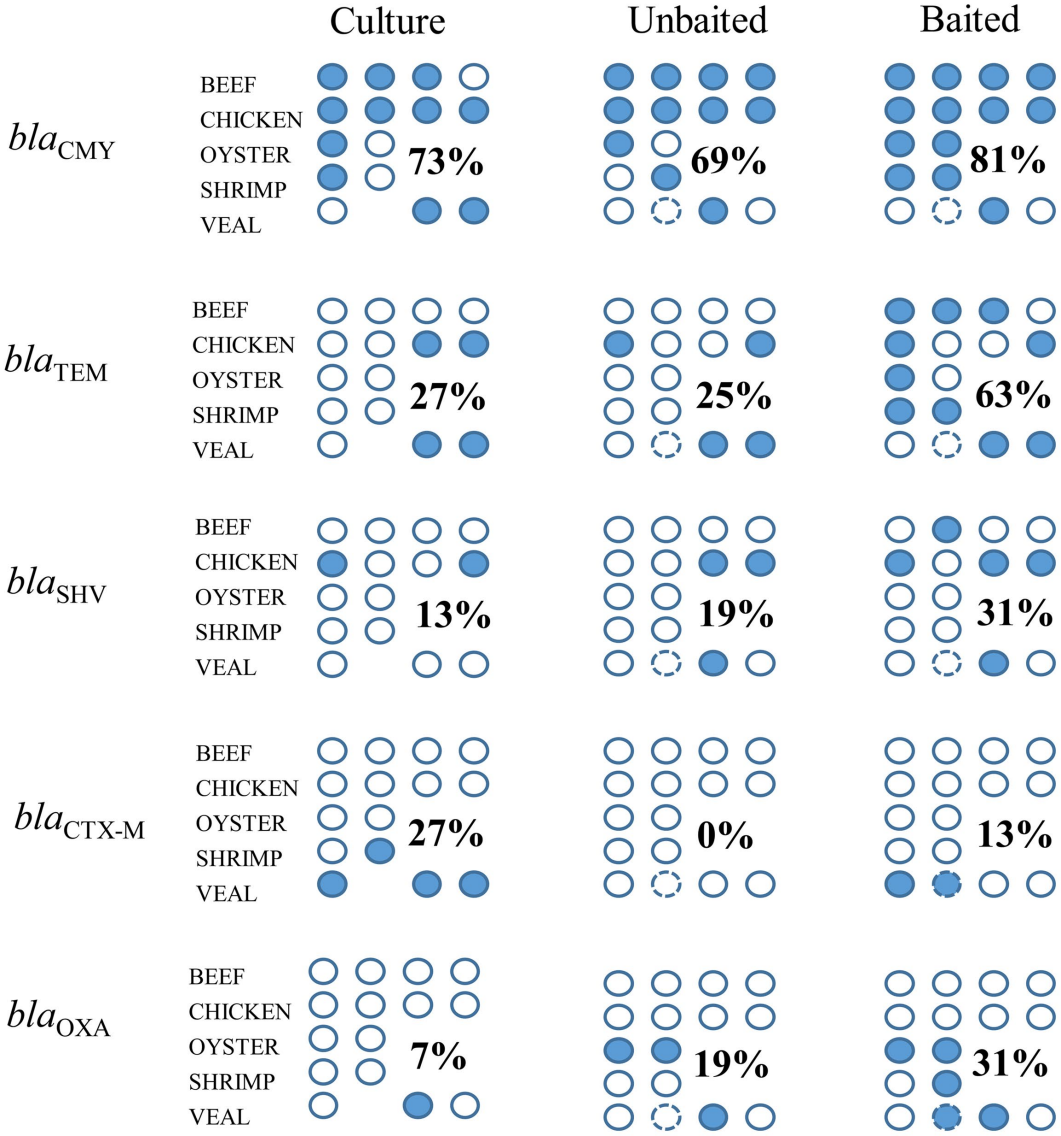
Detection of priority beta-lactamase genes using unbaited, and baited sequencing approaches and comparison to a culture method. Each circle indicates a retail meat sample, filled circles indicate a positive detection in the sample. Numbers indicate the positivity rate of gene detection for each method across commodities (number of positive detections/number of samples). Circle with the broken outline indicated the technical replicate of veal sample 1.

In a previous work, we described the isolation of twenty-eight 3GC resistant *Enterobacteriaceae* strains from the beef, chicken, and veal samples studied here (Rao et al., 2021). Using similar procedures on the oyster and shrimp samples of this study, four additional 3GC resistant *Enterobacteriaceae* strains were obtained. The resistance gene profiles of the meat samples based on the AMR genes present in these 32 isolates are shown in Figure 7. Beta-lactam resistance was most prevalent among the isolates and the priority resistance genes, *bla*_CMY_, *bla*_TEM_, *bla*_SHV_, *bla*_CTX-M_, and *bla*_OXA_ were identified in several. A comparison of the culture-based, unbaited, and baited sequencing approaches showed that the baited data set had the most positive detections for the priority beta-lactamase genes (Figure 8). Using the baited approach, all 16 meat samples were identified as positive for at least one priority beta-lactamase compared to 13 samples for both the culture-based and unbaited sequencing approach. Of particular note was *bla*_TEM_, which had a positivity rate of 63% using the baited dataset compared to the culture method (27%) and unbaited dataset (25%). *bla*_CMY_, *bla*_SHV_, and *bla*_OXA_ also had a higher positivity rates using the baited dataset compared to the other two approaches. *bla*_CTX-M_ was an exception, and a higher positivity rate (27%) using the culture-based method compared to unbaited (0%) and baited (13%) sequencing.

## 4. Discussion

With metagenomic sequencing, the entire resistome of food samples can be characterized within a single experiment. The routine use of this technology would allow food testing laboratories to expand the scope of their AMR monitoring activities. However, metagenomics is expensive, often requiring multiple rounds of in-depth sequencing to detect genes of low abundance. Even so, genes of public health interest may still go undetected. Here, we show that a targeted approach (bait-capture) is a viable alternative to whole genome shotgun metagenomics for the detection of antibiotic resistance genes in meat and seafood enrichments. Across all of the tested foods, the baited libraries required fewer reads (by approximately 40-fold) to detect a higher number of AMR gene family classes, AMR gene families, and plasmid genes compared to the unbaited libraries. These results are similar to others reporting higher recovery of AMR genes from fecal or wastewater samples when using bait-capture supporting the use of this technique for the detection of low abundance genes (Noyes et al., 2017; Lanza et al., 2018; Guitor et al., 2019).

The most significant increases in AMR gene detection by bait capture were seen in the samples that had the fewest unbaited reads map to the AMR database. This implies that the baiting procedure was especially useful for the detection of low abundance genes. This utility was also apparent when comparing the most abundant AMR classes among the commodities between the two datasets. There was good agreement with the most abundant AMR classes, but the relationship did not hold with the less abundant genes. These differences at the lower end of the abundance scale did not impact the AMR gene diversity between the two datasets as would be expected given the minimal contribution of low abundance genes to the overall composition of the resistome (Noyes et al., 2017).

Comparing the AMR genes identified in the baited and unbaited datasets, there was no association of specific genes with either dataset. This observation suggests that no genes were favored by either method. Rather, gene detection varied from sample to sample and likely reflected differences in individual microbiomes and the abundance of the AMR genes therein (Andersen et al., 2016). Genes with a higher frequency of detection, for example *bla*_CMY_, had similar positivity rates across methods, whereas lower abundance genes (*bla*_TEM_, *bla*_SHV_) were more frequently detected in the baited dataset. The few instances where genes were identified in only the unbaited datasets suggest some optimization may be required when implementing the bait-probe library to ensure target genes are being captured. These improvements could involve optimizing bait-probe hybridization or adding additional rounds of sequencing.

A potential drawback of using bait-probe libraries for AMR detection is that the analysis is limited to known resistance genes and prevents the detection of new targets. Screening for plasmid genes is one way to identify samples potentially harboring novel resistance genes, since multi-drug resistance (MDR) plasmids often carry more than one resistance gene. Samples testing positive for MDR plasmids can then be examined more carefully for the presence of AMR bacteria. However, based on our data, more refinements need to be made to the bait-probe library to make this approach more informative. For example, targeting known MDR plasmids, or clinically relevant mobile elements can aid in the identification of samples carrying novel resistance genes or cassettes.

From an AMR perspective, bacterial populations that harbor ESBL genes are important components of the meat microbiome. Of the three methods tested, bait-capture was able to identify the most ESBL positive samples. It may be possible that some of the positives detected by bait-capture may have been due to the presence of DNA in the sample, rather than from viable cells. It is also possible that the sensitivity of bait-capture is higher than plating on selective agar due to the presence of viable but non-culturable cells, silent (unexpressed) AMR genes, or other technical reasons (Deekshit and Srikumar, 2022; Yadav et al., 2022). One notable discrepancy between the culture method and sequencing was the underrepresentation of the *bla*_CTX-M_ gene family in the sequenced datasets. This observation was also noted previously in a study using human fecal samples (Bengtsson-Palme et al., 2015). Like many beta-lactam genes, members of the *bla*_CTX-M_ family comprise a large family of hundreds of alleles (D'Andrea et al., 2013). It is not known why this gene was not detected when other similarly large gene-families from the beta-lactamase class were readily identified by sequencing (*bla*_CMY_, *bla*_TEM_, *bla*_SHV_, *bla*_OXA_). Future iterations of the bait-probe library will need to focus on the improvement of *bla*_CTX-M_ detection since this family is an important, prevalent cause of resistance to third-generation cephalosporins (Castanheira et al., 2021).

**Figure 9:**
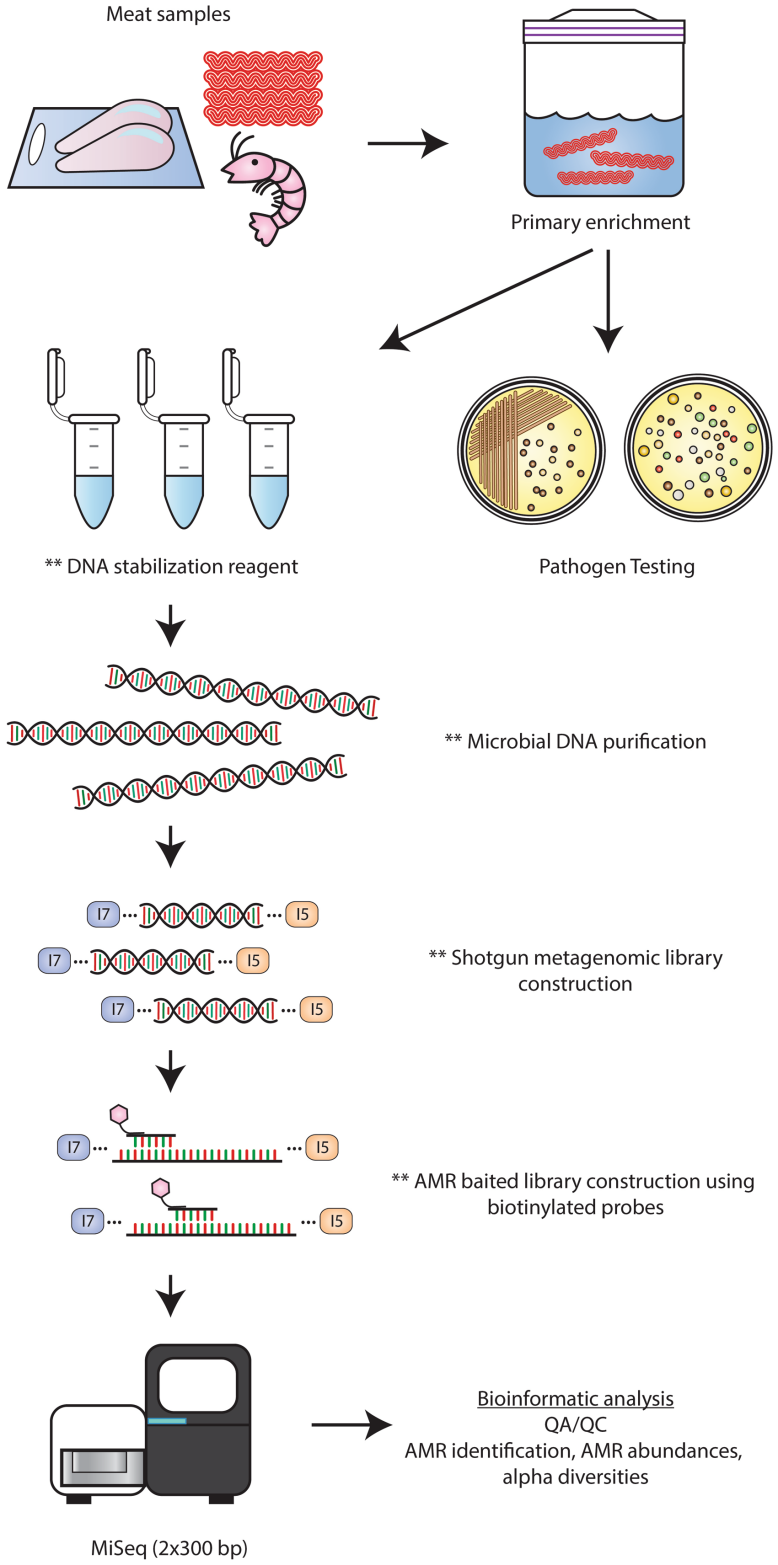
Workflow for AMR detection by bait-capture sequencing. Retail meat microbiomes are cultured in enrichment media. Following sample enrichment, the primary cultures are used for pathogen testing. At this time, a portion of the primary culture can be placed in a DNA preservative solution for downstream bait-capture. After microbial DNA extraction, shotgun metagenomics libraries are constructed. AMR baited libraries are constructed by hybridizing biotinylated AMR probes to shotgun library DNA. Streptavidin beads are used to separate the probe-hybridized target DNA followed by low cycle PCR amplification. Baited libraries are sequenced on an Illumina Miseq platform using 2 × 300 bp chemistry followed by bioinformatics analyses to identify the AMR profiles of each sample. ** denote stopping points where samples can be stored at 4°C or −20°C.

In describing and comparing the results of the baited and unbaited datasets, several observations were made about the AMR and plasmid gene content of retail beef, chicken, oysters, shrimp, and veal. However, given the small sample size of our study, no significant trends were noted as there was a high degree of variation between samples of the same commodity. To make any firm conclusions on the nature of the resistome in Canadian retail meat, more samples, preferably collected as part of national surveillance programs, would need to be analyzed using the bait capture methodology. Similar to the approach used here, regulatory testing workflows for pathogen detection often incorporate a primary enrichment step to encourage the growth of target bacterial populations. Thus, a bait-capture approach can be integrated after primary enrichment to enable routine monitoring of foodborne AMR genes in the same target populations of foodborne bacteria (Figure 9).

## Data availability statement

The datasets presented in this study can be found in online repositories. The names of the repository/repositories and accession number(s) can be found below: https://www.ncbi.nlm.nih.gov/genbank/, BioProject ID PRJNA909287.

## Author contributions

AF: sequencing methodology, investigation, data curation, and original draft preparation. MR: investigation. AC: concept, bait library design, and construction. KW: sequencing methodology. CC: concept and supervision. ST: concept, supervision, original draft preparation, and funding acquisition. All authors contributed to manuscript revision, read, and approved the submitted version.

## Funding

This work was funded by the Government of Canada Shared Priority Project, Genomics Research and Development Initiative (GRDI)-AMR.

## Conflict of interest

The authors declare that the research was conducted in the absence of any commercial or financial relationships that could be construed as a potential conflict of interest.

## Publisher’s note

All claims expressed in this article are solely those of the authors and do not necessarily represent those of their affiliated organizations, or those of the publisher, the editors and the reviewers. Any product that may be evaluated in this article, or claim that may be made by its manufacturer, is not guaranteed or endorsed by the publisher.

## Footnotes

^1^ https://github.com/lh3/seqtk
