# Supplementary material for: Targeted metagenomics using bait-capture to detect antibiotic resistance genes in retail meat and seafood
Source: Front Microbiol. 2023 Jul 13;14:1188872. doi: 10.3389/fmicb.2023.1188872 (PMC10373929; doi:10.3389/fmicb.2023.1188872)
Supplement: Supplementary file 1 [file Table_1.pdf]

Supplementary Material

Targeted metagenomics using bait-capture to detect antibiotic resistance genes in retail meat and seafood

Annika Flint[1], Ashley Cooper[2], Mary Rao[1], Kelly Weedmark[1], Catherine Carrillo[2], and Sandeep Tamber[1,*]

*** Correspondence:**
Sandeep Tamber
sandeep.tamber@hc-sc.gc.ca

Table S1. AMR classes identified in the unbaited (u), baited (b), or both (x) datasets

AMR class	Beef				Chicken				Oyster		Shrimp		Veal			
	1	2	3	4	1	2	3	4	1	2	1	2	1	1 TR	2	3
Beta-lactam	x	x	x	x	x	x	x	x	x	x	x	x	x	x	x	x
Tetracycline	x	b	b	x	x	x	x	x	x	x	x	x	x	x	x	x
Aminoglycoside	x	b	x	b	x	x	x	x	x	x	x	b	b	x	x	x
Phenicol	x	x	b		x		x	x	x	b	b	x	b	b	x	x
Quaternary Ammonium	x	b			x	x	x	b	b	b	x	b			x	x
Sulfonamide	b	b	b		x	x	x	x	b		b	b			x	x
Quinolone	x	x	x	x					x	x	x	x		b	x	
Fosfomycin	x	x		x		x	x		x	b	x	b			x	
Trimethoprim	b		b		x	x			b		b				x	x
Lincosamide-Streptogramin		b	b	b	x	b		b			x	x			b	
Streptothricin					x	x					b		b	b	x	x
Macrolide					x	b					b	b			b	x
Lincosamide					x			b			x	b			b	
Phenicol-Quinolone		x		x		u		x	b						x	
Streptogramin					x	b	x								b	
Glycopeptide					x						x	b				
Bleomycin							x					b			x	
Pleuromutilin					x						b	x				
Fusidic acid											b	b				
Phenicol/oxazolidinone											b					
Total (unbaited)	7	4	3	5	14	9	8	6	6	4	7	5	2	3	12	8
Total (baited)	9	10	8	7	14	11	9	9	10	7	17	15	5	6	16	9

Table S2. Most frequently detected AMR gene classes per commodity (% rel abundance)

**Rank**	**Beef**		**Chicken**		**Oyster**		**Shrimp**		**Veal**	
	Unbaited	Baited	Unbaited	Baited	Unbaited	Baited	Unbaited	Baited	Unbaited	Baited
**1**	**BL (67)**	**BL (43)**	**AG (45)**	**AG (46)**	**BL (51)**	**BL (38)**	BL (30)	AG (26)	**AG (33)**	**AG (32)**
**2**	QUIN (8)	AG (21)	**BL (21)**	**BL (16)**	QUIN (14)	AG (13)	GLY (18)	BL (18)	**BL (19)**	**BL (18)**
**3**	FOS (6)	PHEN (6)	**TET (7)**	**TET (7)**	AG (10)	QUIN (10)	TET (15)	PHEN (9)	**PHEN (13)**	**PHEN (11)**
**4**	AG (4)	TET (6)	**SUL (5)**	**SUL (4)**	**TET (10)**	**TET (10)**	LS (6)	MAC (8)	**TET (12)**	**TET (11)**
**5**	PHEN (4)	TMP (5)	GLY (3)	QAC (4)	FOS (7)	QAC (8)	QUIN (6)	TET (8)	SUL (6)	SAT (6)
**6**	TET (4)	QUIN (4)	SAT (3)	MAC (4)	PHEN (3)	FOS (7)	LIN (6)	GLY (6)	QAC (5)	TMP (6)
**7**	PQ (4)	SUL (4)	QAC (2)	GLY (3)	EFF (3)	PHEN (5)	FOS (6)	TMP (4)	SAT (4)	QAC (5)
**8**	QAC (2)	FOS (3)	MAC (2)	SAT (3)	(QAC (0)	TMP (5)	AG (3)	LS (3)	TMP (4)	SUL (4)
**9**	TMP (0)	LS (3)	PHEN (2)	LS (2)	TMP (0)	SUL (3)	PHEN (3)	QUIN (3)	PQ (1)	MAC (2)
**10**	SUL (0)	PQ (2)	TMP (2)	LIN (2)	SUL (0)	PQ (2)	**QAC (3)**	**QAC (3)**	MAC (1)	QUIN (2)

Rankings that are similar between baited and unbaited datasets are shown in **bold**.
